# Xdrop: Targeted sequencing of long DNA molecules from low input samples using droplet sorting

**DOI:** 10.1002/humu.24063

**Published:** 2020-06-29

**Authors:** Esben B. Madsen, Ida Höijer, Thomas Kvist, Adam Ameur, Marie J. Mikkelsen

**Affiliations:** ^1^ Samplix Herlev Denmark; ^2^ Science for Life Laboratory, Department of Immunology, Genetics and Pathology Uppsala University Uppsala Sweden; ^3^ Department of Epidemiology and Preventive Medicine Monash University Melbourne Australia

**Keywords:** long‐read sequencing, nanopore sequencing, SMRT sequencing, targeted enrichment, targeted sequencing, Xdrop

## Abstract

Long‐read sequencing can resolve regions of the genome that are inaccessible to short reads, and therefore are ideal for genome‐gap closure, solving structural rearrangements and sequencing through repetitive elements. Here we introduce the Xdrop technology: a novel microfluidic‐based system that allows for targeted enrichment of long DNA molecules starting from only a few nanograms of DNA. Xdrop is based on the isolation of long DNA fragments in millions of droplets, where the droplets containing a target sequence of interest are fluorescently labeled and sorted using flow cytometry. The final product from the Xdrop procedure is an enriched population of long DNA molecules that can be investigated by sequencing. To demonstrate the capability of Xdrop, we performed enrichment of the human papilloma virus 18 integrated into the genome of human HeLa cells. Analysis of the sequencing reads resolved three HPV18‐chr8 integrations at base‐pair resolution, and the captured fragments extended up to 30 kb into the human genome at the integration sites. Further, we enriched the complete *TP53* locus in a leukemia cell line and could successfully phase coexisting mutations using PacBio sequencing. In summary, our results show that Xdrop is an efficient enrichment technology for studying complex genomic regions.

## Graphical abstract

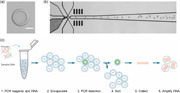

## 1 INTRODUCTION

Short‐read next‐generation sequencing (NGS) technologies are based on the generation of millions of DNA sequence reads, typically 100–500 bp in length. As the sequence information is broken up in millions of short sequence fragments, it is crucial that the reads are assembled correctly in order not to lose sequence information. This is a particularly a challenging task in regions with repetitive sequences or with structural rearrangements (Alkan, Coe, & Eichler, 2011). Long‐read single‐molecule sequencing technologies provided by Pacific Biosciences (PacBio) and Oxford Nanopore Technologies (ONT) are capable of reading DNA molecules in a range between tens to hundreds of kilobases, thereby practically solving the issue with assembling the sequence reads correctly, even for organisms with large genome sizes, such as humans (Jain et al., 2018; Seo et al., 2016). Although long‐read technologies have seen a dramatic increase in throughput during the last few years, leading to a cost per base approaching that for short‐read sequencing, it is still relatively expensive to generate whole‐genome coverage at a depth sufficient for detailed genome analysis. The cost can be reduced by enriching the DNA sample for the genomic regions of interest and thereby increasing the number of reads covering those specific regions.

At present, there is a wide selection of target enrichment methods for short‐read sequencing technologies (reviewed in Mamanova et al., 2010), but only a few that are compatible with the long‐read sequencing platforms. The most common approach to enrich for long DNA fragments is to perform a long‐range polymerase chain reaction (LR‐PCR) followed by sequencing of the resulting amplicons (Ardui et al., 2017; Lode et al., 2018). However, it is technically challenging to amplify regions longer than 10 kb using LR‐PCR and both ends of the target fragment must be known to design the PCR primers. An alternative approach is to perform hybridization (Wang et al., 2015) using short DNA‐ or RNA probes that are used to pull down the target of interest. Hybridization‐based methods can enrich for fragments up to 10 kb based on the information of a short probe (<200 bp) but require relatively large amounts of input DNA (>500 ng). Both long‐range PCR and hybridization methods also have a risk of introducing chimeric reads during amplification steps in the sample or library preparation. Alternative protocols have recently been developed that uses the CRISPR/Cas system and do not require any PCR amplification step (Gabrieli, Sharim, Michaeli, & Ebenstein, 2017; Gießelmann et al., 2018; Gilpatrick et al., 2019; Stangl et al., 2019; Tsai et al., 2017). The amplification‐free enrichment makes it possible to interrogate genomic regions that are difficult to amplify, such as expanded repeats (Gießelmann et al., 2018; Hoijer et al., 2018; Tsai et al., 2017), but require high amounts of input DNA and are relatively labor‐intensive. Thus, there is a need for a method capable of isolating long‐fragments from small amounts of input DNA where only a small piece of the sequence is known, using a standardized and fast protocol.

Here we present the Xdrop technology, a novel microfluidic‐based system that allows for targeted enrichment of long DNA fragments. The Xdrop system permits the enriched DNA to be sequenced on a long‐read sequencing platform, such as PacBio or ONT, or alternatively to provide long‐fragment information from short‐read sequencing. Only ~150 bases of sequence information are needed to perform enrichment of DNA fragments of up to 40 kb in size, and the protocol requires as little as 1–2 ng of DNA as input to enrich for a specific region in a human‐sized genome. All amplification reactions are performed in small droplets and this procedure eliminates competition between amplification reactions and minimizes the risk of intermolecular chimeric molecules being formed. As a proof‐of‐principle, we applied the Xdrop technology to determine the integration sites of human papillomavirus 18 (HPV18) in the HeLa cell line. We further demonstrated the utility of this technology by enriching for the cancer suppressor gene *TP53* in a human leukemia cancer cell line, allowing for detection and phasing of mutations.

## 2 METHODS

### 2.1 PCR and droplet chemicals

For HPV18 enrichments HeLa DNA (Thermo Fisher Scientific) was diluted with DNAase free water (Gibco) to 0.5 ng/µl before use. PCR‐mix for 20 µl was set up as follows: 2 µl of 10 PCR‐buffer without detergent (Thermo Fisher Scientific), 2 µl 25 mM MgCl_2_ (Thermo Fisher Scientific), 2 µl 2 mM dNTP (Thermo Fisher Scientific), 1.2 µl glycerol 50%, 0.4 µl GoTaq polymerase (5U/µl) (Promega), 0.25 µl (2 mg/ml) Bovine Serum Albumin (Thermo Fisher Scientific), 0.8 µl HPV18 fw primer (10 µM) 5′‐TGTGCTGGAGTGGAAATTGG‐3′, 0.8 µl HPV18 rev primer (10 µM) 5′‐GGCATGGGAACTTTCAGTGTC‐3′, 0.6 µl HPV18‐TP1 probe (10 µM) 5′‐FAM‐CAACACCTAAAGGCTGACCACGG‐BHQ1‐3′, 1 µl 0.5 ng/µl Hela DNA and water to 20 µl. For the primary droplet production, 3% RAN fluorosurfactant in Novec HFE‐7500 was used as a carrier phase. For the secondary droplets a DE‐buffer containing 1.5× Optima buffer (40 mM Tris‐HCl, 60 mM Trizma‐base, 25 mM (NH_4_)_2_SO_4_, 0.015% Tween 80, and 45 mM NaCl) and 3% glycerol was used as a carrier phase.

For *TP53* enrichments Jurkat DNA (Thermo Fisher Scientific) was diluted with DNAase free water (Gibco) to 4 ng/µl before use. PCR‐mix for 40 µl was set up as follows: 20 μl of 2× dPCR‐mix (Samplix), 0.8 µl TP53 fw primer (10 µM) 5′‐GGTGTGATGGGATGGATAAA‐3′, 0.8 µl TP53 rev primer 5′‐CCCTGCATTTCTTTTGTTTG‐3′, 2 µl 2 ng/µl Jurkat DNA and water to 40 µl. For the primary droplet production dPCR‐oil (Samplix) was used as a carrier phase. For the secondary droplets, dPCR‐buffer (Samplix) was used as carrier phase.

### 2.2 Droplet production

For the HPV18 enrichment, double emulsion droplets were produced using a two‐step emulsification procedure, initially creating water in oil (W–O) droplets followed by second emulsification to create water in oil in water (W–O–W) droplets. For the *TP53* enrichment, an Xdrop cartridge (Samplix) that generates double emulsion in a single step was used in combination with the Xdrop droplet generator instrument (Samplix).

### 2.3 Primary droplets (W–O)

The initial chip used to prepare the primary emulsion was a 14 µm etch depth hydrophobic “Small Droplet Chip, 14 µm” (Dolomite Microfluidics). Liquids were pushed into the microfluidic chip using MFCS‐EZ pressure controller (Fluigent, Germany) applying pressures of 640 mbar on the primary sample (PCR) and 650 mbar to the secondary liquid (Oil). Droplet production was done for ∼40 min, processing a total of 40 µl PCR mixture.

### 2.4 Secondary emulsions (W–O–W)

Immediately following primary production, droplets were collected in PTFE‐tubes using a 1 ml syringe (Scientific Glass Engineering, Australia) ensuring an air‐free liquid system to pull the droplets into the tube. The tube was then connected to the inlet‐position of the 4‐way Linear Connector (Dolomite microfluidics, UK connector; Part number 3000024). Droplets were pushed into the chip at 0.25 µl/min using a Legato 110 syringe pump (KD Scientific). During secondary droplet production, spacer oil was applied into the chip using a syringe system identical to that carrying the droplets, delivering oil to space the introduced droplets before the second emulsification. Spacer oil was connected to position 2 in the connector using a syringe pump set to deliver a flow of 0.40 µl/min. Double emulsion buffer (DE) was introduced to the chip using a Legato 100 (KD Scientific) single syringe pump applying pressure to a 10 ml syringe (Scientific Glass Engineering, Australia). The pump speed of the DE‐buffer was set to 28 µl/min. Second emulsification was performed for 160 min until all primary droplets had passed the junction of the DE‐chip.

### 2.5 Droplet polymerase chain reaction

After droplet generation, the droplets were transferred to PCR‐tubes and put into a thermo‐cycler. The following PCR conditions were used: 94°C for 2 min followed by 40 cycles with 94°C for 3 s and 60°C for 30 s. The PCR ramping‐rate was set to 1.5°C/sec.

### 2.6 Droplet staining

The *TP53* droplets were diluted with 1 ml of dPCR‐buffer (Samplix) and stained with 10 µl Droplet Stain (Samplix) before droplet sorting.

### 2.7 Droplet sorting and gating

Sorting was carried out on a single laser 488 nm, S3e cell sorter (BioRad inc.) using ProSort software (v. 1.3b). Instrument PMTs were adjusted to: FSC = 239, SSC = 261, FL1 = 590, and FL2 = 367. FSC was used as the primary threshold and the value was set to 1.00. The gating of positive droplets was performed in three consecutive gating events. The first gate was set to discriminate between double emulsion droplets and “other” elements in the carrier buffer. The second gate was used to split the double emulsion droplets from the first gate into a fluorescent and nonfluorescent double emulsion droplets. The third gate was applied to ensure that only positive droplets with the expected properties were sorted. Sorting purity was set to “Enrich” and the event rate was kept as close to 4,000 events/s as possible throughout the experiment. Before sorting droplets, 5 µl Tris (10 mM) was placed at the bottom of the 1.5 ml collection tube to avoid disrupting the sorted droplets. Upon completed sorting, the collection tube was centrifuged at 1,000*g* for 10 s to collect any liquid from the side of the tube, arising from the splash impact of sorted droplets hitting the liquid surface of the inside tube.

### 2.8 DNA amplification

The collected droplets were coalesced by adding 20 µl of PicoBreak (Sphere Fluidics), mixing and centrifuging the sample. About 3–10 µl of the resulting aqueous‐phase was used as a template for a multiple displacement amplification (MDA) reaction. The MDA reaction mix was kindly provided by Samplix. For the HPV18 enrichment, the MDA reaction was emulsified on an x‐junction droplet generator chip (ChipShop) using 1% PicoSurf in 7500‐Novec oil as a carrier phase. The droplet production was driven by air pressure controlled by a pressure regulator (Fluigent). For the *TP53* enrichment, the MDA reaction was emulsified on a dedicated dMDA cartridge (Samplix). The droplets containing the MDA reaction were incubated for 16 hr at 30°C followed by 10 min at 65°C to terminate the reaction. About 6 µl from the MDA reaction was used as a template for a second droplet MDA reaction. After each MDA round the emulsions where coalesced using 20 µl PicoBreak.

### 2.9 PacBio sequencing of HPV18 in HeLa cells and TP53 in Jurkat cells

A 2 kb PacBio library was produced from 1 µg HPV18 enriched HeLa DNA using the SMRTbell Template Prep Kit 1.0 according to manufacturer's instructions. The library was sequenced on one SMRTcell on the PacBio RSII instrument using C4 chemistry and P6 polymerase and 240 min movie time. A 2 kb PacBio library was produced from 750 ng HeLa DNA using the SMRTbell Template Prep Kit 1.0 according to manufacturer's instructions. SMRTcell on the PacBio Sequel instrument using v. 2.1 chemistry and 600 min movie time. A 5 kb PacBio library was produced from 1 µg *TP53* enriched Jurkat DNA using the SMRTbell Template Prep Kit 1.0 according to manufacturer's instructions. The library was sequenced on one SMRTcell on the PacBio RSII instrument using C4 chemistry and P6 polymerase and 240 min movie time. CCS reads were generated for all PacBio data sets before downstream analysis.

### 2.10 Illumina sequencing of HPV18 in HeLa cells

The HPV18 enriched sample was diluted 10‐fold with Tris‐HCl to reduce the MgCl_2_ concentration before library preparation. A standard DNA library for Illumina sequencing was performed, developed, and validated by Eurofins Genomics and 5 million read pairs of 2 × 150 bp were generated.

### 2.11 Nanopore sequencing of HPV18 in HeLa cells

An ONT library was produced from 400 ng of HPV18 enriched HeLa DNA using the Rapid Sequencing (SQK‐RAD003) protocol. The library was sequenced on 1 MIN106 flowcell (R9.4) for 17.5 hr with subsequent Albacore base calling (v2.1.3). All was performed using standard settings according to the manufacturer's recommendations.

### 2.12 Detection of HPV18 integration sites

CLC Genomic Workbench (Qiagen) was used for the bioinformatic analysis of Illumina data and MiniMap2 for the PacBio and ONT data. HPV18 integration sites were identified by mapping all sequence reads to the HPV18 reference genome. The reads mapping to HPV18 was subsequently re‐mapped to the human reference genome GRCh38 (hg38). Reads mapping to both genomes were considered HPV18/Chr8 fusion‐reads. For the Illumina data the mapping settings were as follows: Match Score: 1, Mismatch cost: 2, Linear gap cost, Insertion cost: 1, Deletion cost 3, Insertion open cost: 6, Deletion open cost: 6, Deletion extend cost: 1 Length Fraction: 0.8, Similarity Fraction: 0.9. For the PacBio and ONT mapping, MiniMap2 standard settings were used.

### 2.13 Validation of HPV18 integration sites through sanger sequencing

PCR primers located on each side of the HPV18 fusion points were designed and used to generate PCR amplicons across the fusion point. These PCR products were Sanger sequenced using one of the PCR primers.

### 2.14 Detection and phasing of TP53 mutations

CCS reads were mapped to the human reference genome GRCh38 using CLC Genomic Workbench. Mapping settings were as follows: Match Score: 1, Mismatch cost: 2, Linear gap cost, Insertion cost: 1, Deletion cost 3, Insertion open cost: 6, Deletion open cost: 6, Deletion extend cost: 1 Length Fraction: 0.5, Similarity Fraction: 0.8. Previously known *TP53* mutations were detected and phased by manual analysis of sequences using the Integrative Genomics Viewer 2.5.2 (IGV). Only CCS reads that spanned both mutations sites were used for phasing of the mutations.

## 3 RESULTS

### 3.1 Production of millions of double emulsion droplets using the Xdrop system

**Figure 1:**
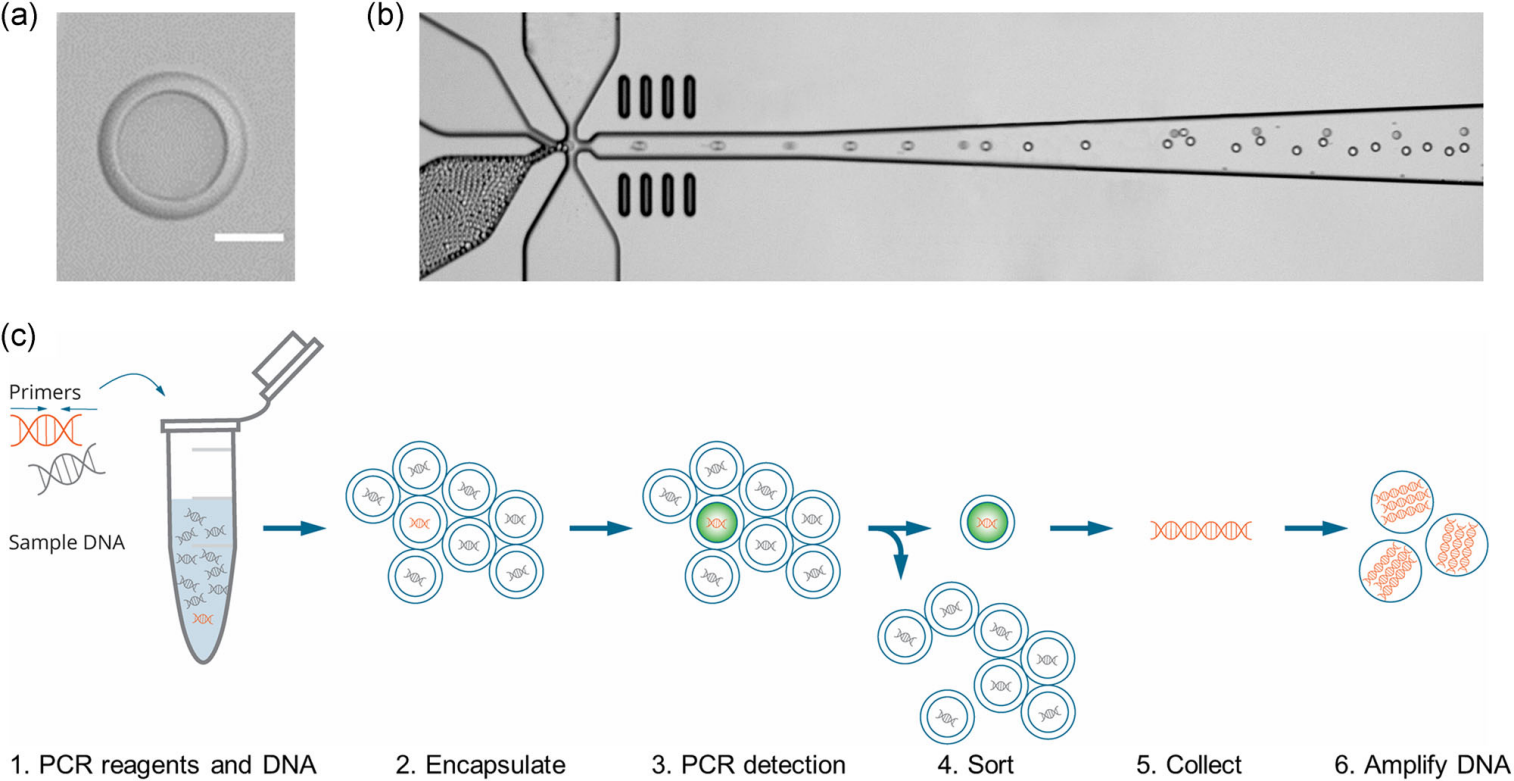
Overview of the Xdrop technology. (a) Close‐up of a double emulsion (DE) droplet. The inner droplet contains PCR‐reagents and sample DNA and is surrounded by a thin oil shell. The scalebar is 10 μm. (b) High‐speed camera photo of DE droplet generation on chip. Water‐in‐oil droplets are being injected on the left, to generate water‐in‐oil‐in‐water droplets at the x‐junction. (c) Overview of Xdrop enrichment workflow. PCR reagents including primers are mixed with sample DNA (1) before being encapsulated in DE droplets (2). Droplet PCR allows fluorescence‐based detection of the DNA molecules of interest (3) that are then sorted out on a cell sorter (4). The DNA from the sorted droplets is finally collected (5) and amplified using droplet MDA. The orange DNA helixes depict the target DNA of interest and gray DNA helixes depict nontarget DNA. MDA, multiple displacement amplification; PCR, polymerase chain reaction

The Xdrop technology is based on the generation of millions of double emulsion (DE) water‐in‐oil‐in‐water droplets with an inner diameter of 15 µm and outer diameter of 20 µm (see Figure 1a), that is in the same size‐range as large eukaryotic cells. The DE droplet generation is performed using a microfluidic chip that has the capacity of producing up to 3,000 droplets per second (Figure 1b). A high‐speed camera imaging of the DE droplet production is available as the Supporting Information material. Because of their small volume and high stability, the DE droplets are ideal for the Xdrop enrichment work flow. The material required for an Xdrop enrichment assay is a genomic DNA sample, millions of DE droplets, and a set of PCR primers that uniquely amplify a small piece of DNA (100–200 bp) within the target region of interest.

### 3.2 Isolation of DE droplets containing long DNA molecules of interest

Before DE droplet generation, sample DNA is mixed with PCR reagents, target‐specific PCR primers, and, depending on the detection chemistry, a hydrolysis probe (Figure 1c). DNA and PCR‐reagents are then partitioned in 20–34 million of picoliter DE droplets, of which the majority will only contain either a single DNA molecule or none. The droplets are thermocycled in a standard PCR apparatus, allowing 20–34 million individual PCR‐reactions to be performed in parallel. In the droplets containing a target DNA molecule, the target‐specific primers and hydrolysis probe will bind to the template and, upon primer extension, the 5′ exonuclease activity of the polymerase cleaves the fluorophore from the probe thereby releasing a fluorescent dye. Alternatively, the droplets containing a PCR product can be identified by staining the droplets with a DNA‐intercalating fluorescent dye. By detecting the fluorescent droplets, it is, therefore, possible to identify and isolate the droplets carrying the DNA molecules of interest (Figure 1c). The detection and sorting can be carried out on a standard laboratory cell sorter (FACS), as the carrier fluid of the DE droplets is aqueous and the DE droplets are sufficiently small. On the FACS apparatus, the DE droplets scatter the light and are easy to distinguish from oil‐in‐water droplets using forward‐ and side‐scatter. In a second gate, the PCR positive DE droplets with high FITC/FL1 fluorescence are separated from the PCR negative droplets that are low in FITC/FL1 fluorescence. The droplets containing the target DNA molecule of interest are collected (Figure 1c) and coalesced to release the DNA into solution. As the amount of DNA fragments collected from the sorted droplets is in the femtogram range and hence too low to be sequenced using NGS technologies, the sorted DNA fragments are amplified using Phi29 polymerase multiple displacement amplification (MDA). To avoid amplification bias and the introduction of chimeric amplification products, the DNA and MDA reaction mix is emulsified, creating tens of thousands of separate MDA reactions droplets. Only a fraction of the droplets will contain a DNA molecule in the first round of amplification. Therefore, the MDA emulsion is coalesced and used as a template for the second round of droplet MDA (dMDA) to generate a sufficient amount of DNA.

### 3.3 Xdrop enrichment for integrated HPV18 virus

To evaluate the Xdrop enrichment procedure on a human sample with biological relevance, we designed an enrichment experiment targeting human papilloma virus 18 (HPV18) integrated into the HeLa cell line. Previous studies have shown that the HeLa genome contains integrated HPV18 virus DNA (Adey et al., 2013; Schneider‐Gadicke & Schwarz, 1986). A single primer‐probe set located between HPV18 position 5916 and 6014 was designed to detect HPV18 containing DNA fragments. About 1.5 ng of genomic HeLa DNA, corresponding to 417 HeLA genome copies, was partitioned into DE droplets before amplification with the target‐specific primers. The HPV18 positive DE droplets with high FITC/FL1 fluorescence were easily separated from the HPV18 negative DE droplets on the scatter plot obtained from the FACS sorting (Figure S1A). A total of 143 HPV18 positive DE droplets were sorted and the collected HPV18 DNA fragments were amplified using dMDA. After two rounds of dMDA, the total DNA content was 3 µg with an average DNA fragment length of more than 15 kb (Figure S1B). The number of HPV18 containing DNA fragments was estimated using a semiquantitative qPCR and was found to be ∼1,000‐fold higher per ng of total DNA as compared to the starting material.

### 3.4 Detection of HPV18 integration sites in the HeLa genome

**Figure 2:**
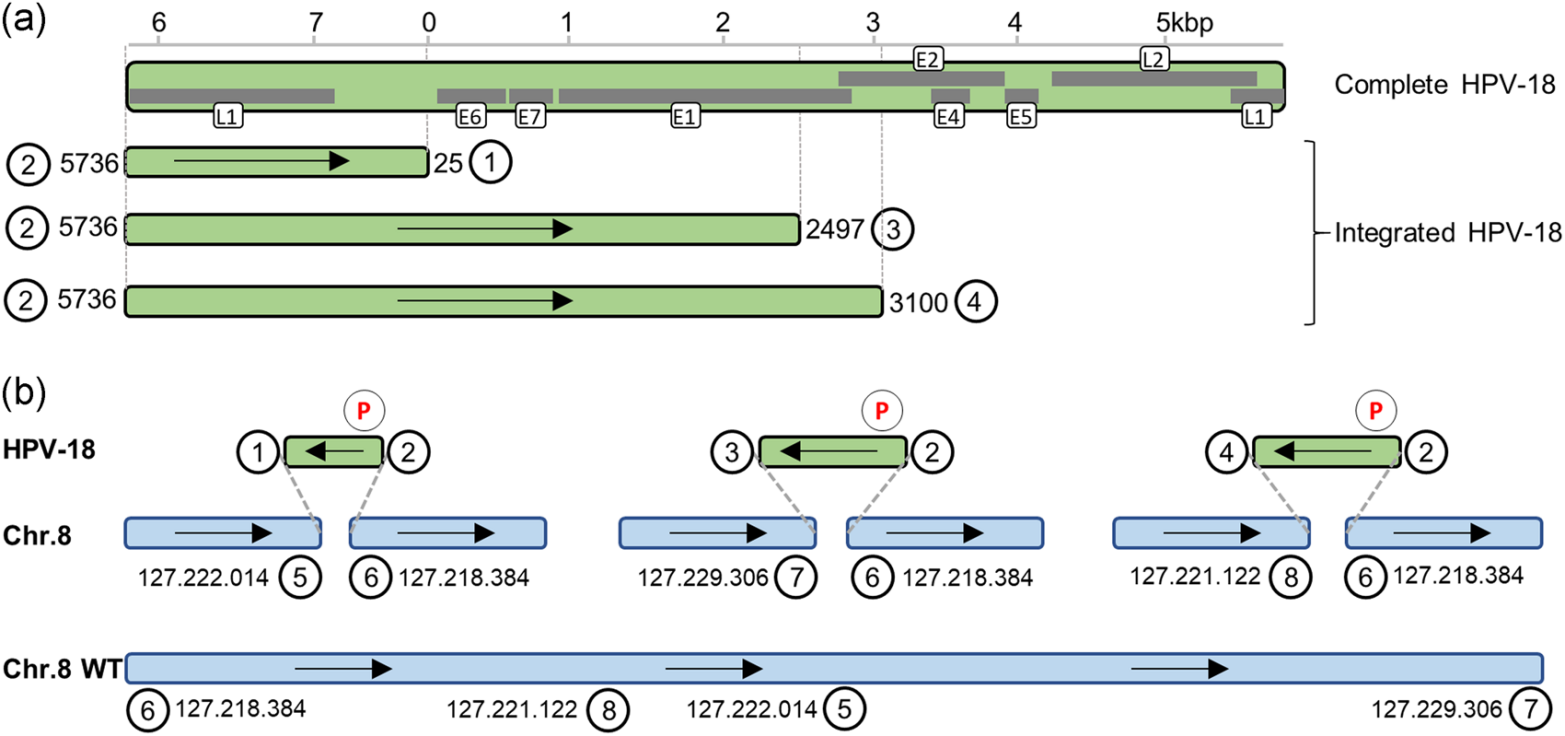
Detection of HPV18 integration sites by Xdrop enrichment and SMRT sequencing. (a) Overview of the complete HPV18 genome with genes depicted in gray. Below the three types of integrated HPV18 found in the HeLa genome. The breakpoints are shown with numbered circles. (b) Overview of the HPV18 fusion points and the suggested structure of the integrations identified in the HeLa genome. The positions of the chromosome 8 integration sites refer to the GRCh38 genome assembly. The position of the primers used for enrichment is shown with circled P's. The numbered circles correspond to the numbering in a. SMRT, single‐molecule real‐time

To generate long continuous reads of the enriched molecules, we performed single‐molecule real‐time (SMRT) sequencing. A 2 kb insert library was prepared directly from the HPV18 enriched DNA sample and sequencing was performed on the PacBio RSII system. Our rationale for choosing a relatively short library size (2 kb) in this experiment was to generate high accuracy circular consensus sequences (CCS; Travers, Chin, Rank, Eid, & Turner, 2010) of a sufficient length to easily identify the HPV18 integration sites. The sequencing resulted in 41,402 CCS reads, of which 688 reads (1.7%) could be mapped to the HPV18 viral genome. As a control, we performed sequencing of unenriched HeLa DNA on the higher throughput PacBio Sequel system, resulting in only 5 of 294,284 CCS reads (0.0017%) mapped to HPV18. Based on these numbers we estimate that Xdrop gives a ~1,000‐fold enrichment of HPV18. The HPV18 reads were covering the HPV18 gene region E1, E6–E7, and L1 but lacked the central part of the viral genome encoding E2, E4, E5, and L2 (Figure 2a and Figure S2). This is in concordance with the structure previously reported for HPV18 integrated in the HeLa genome (Adey et al., 2013). By extracting all reads mapping to HPV18 and realigning them to the human reference genome, we identified four HPV18 fusion points to a region of chromosome 8 (Figure 2b). Detailed analysis revealed that one end of HPV18 is fused to three different locations in chromosome 8, while the other end of HPV18 (position 5736) always is fused to the same position on chromosome 8. The proposed structure of the HPV18 integrations in the HeLa genome is shown in Figure 2b. All of the identified HPV18‐HeLa fusion points were validated by PCR and Sanger sequencing (Figure S3).

To evaluate the robustness of the Xdrop method, we repeated the HPV18 enrichment from the same HeLa DNA sample and performed sequencing on Illumina and ONT instruments. The same HPV18/Chr8 fusion points and all four fusion sites were also identified using these technologies. The coverage at each fusion point is shown in Table S1, and importantly the enriched DNA fragments extend up to 30 kb into the human genome at the integration sites (Figure S4). The percentage of on‐target reads, including the reads covering the integration site region, was 5%, 4%, and 2% for the PacBio, ONT, and Illumina data set, respectively (Table S2). Our sequencing results demonstrate that the Xdrop system is able to enrich for very long DNA molecules and that different types of sequencing libraries can be produced from the resulting enriched DNA.

### 3.5 Detection and phasing of TP53 mutations in human cancer cell line

**Figure 3:**
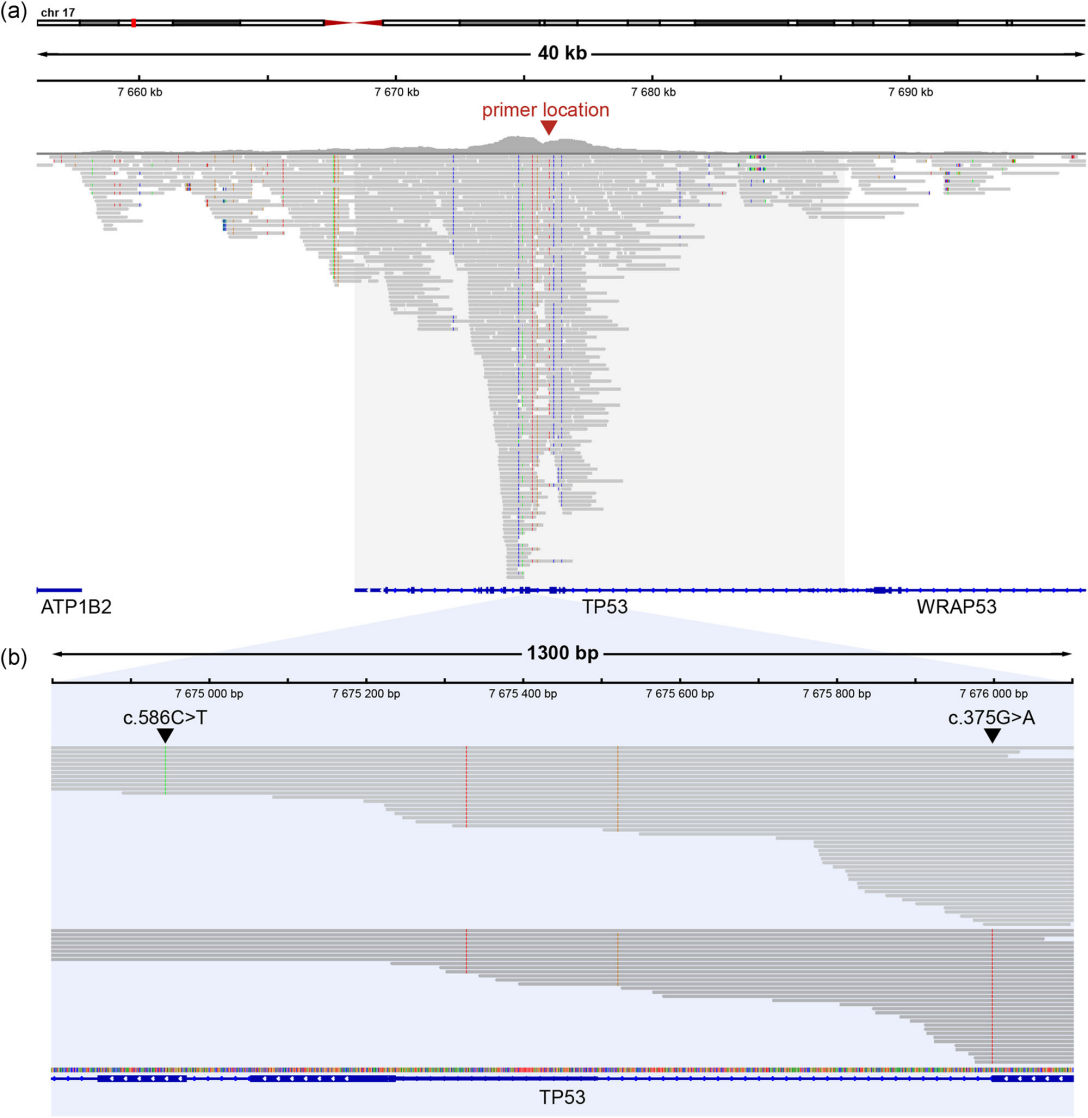
Xdrop enrichment and SMRT sequencing of *TP53* for mutation detection and phasing. (a) IGV image of CCS reads mapped to the *TP53* locus. The location of the detection primers is marked by the red arrow. The gray box highlights the *TP53* gene. (b) IGV image showing the phasing of *TP53* mutations. Black arrows mark the mutation sites for NG_017013.2:g.16557G>A and NG_017013.2:g.17606C>T. SMRT, single‐molecule real‐time

To demonstrate the versatility of the Xdrop enrichment technology, we designed detection primers to enrich the cancer suppressor gene *TP53* in the human Jurkat leukemia T cell line. In addition, we used a DNA intercalating dye for the PCR detection instead of a hydrolysis probe. The primers were located within intron 4 (Figure 3a), which is a relatively conserved region within this otherwise highly mutation prone gene (Bouaoun et al., 2016). About 4 ng of genomic DNA was partitioned in DE droplets and used in the Xdrop enrichment. After droplet PCR, FACS sorting and dMDA we obtained ~1 µg of DNA with an average size of 8 kb. The enriched DNA was sequenced using SMRT sequencing on a RSII system with a 5 kb insert size library. With this relatively small insert size, we increase the proportion of high‐quality CCS reads, which are of importance for detecting SNVs confidently. Out of the 66,183 CCS reads generated, 55,496 reads mapped to the human reference (GRCh38) and 655 reads (1.18%) mapped to the *TP53* locus, spanning a >40 kb region (Figure 3a). The average read length of the CCS reads at the *TP53* locus was 1.9 kb and the longest CCS read >11 kb. The Jurkat cell line has two previously described cancer mutations in *TP53*, NG_017013.2:g.16557G>A and NG_017013.2:g.17606C>T, which we were able to detect in our data. The coverage at these mutation sites was 77x and 103x, respectively. Several of the reads (*n* = 17) spanned both mutation sites. Based on information from these reads, we were able to confirm that NG_017013.2:g.16557 G>A and NG_017013.2:g.17606C>T are present on different alleles in Jurkat cells (Figure 3b).

## 4 DISCUSSION

We have demonstrated the Xdrop targeted enrichment technology on two different applications. First by sorting out DNA fragments containing integrated HPV18 virus and using a FAM‐labeled hydrolysis probe for the detection of droplets containing HPV18 DNA molecules. Second, we sorted out DNA fragments containing the *TP53* gene region using a DNA‐intercalating dye. Multiplexing of several different DNA targets by adding additional primer‐ and probe‐sets is feasible. The targets could then be distinguished by differently labeled probes, thereby allowing quantification of multiple targets in the same sample, which would be useful for example to measure copy number variability. Alternatively, the different targets could be detected and sorted using the same intercalating dye, which might be preferable for example when tiling over a longer genomic region. It is also possible to enrich for the same region in several samples and perform a multiplexed sequencing run, by introducing sample‐specific barcodes into the library before sequencing. By barcoding of Xdrop enriched samples, the cost of targeted long‐read sequencing can be reduced significantly.

The level of enrichment by Xdrop technology is comparable to other long‐read targeted enrichment strategies (Gabrieli et al., 2017; Gilpatrick et al., 2019; Tsai et al., 2017). Most of the targeted enrichment methods developed for single‐molecule long‐read technologies utilize the CRISPR/Cas9 system for excision of the region of interest, and they require micrograms of input DNA (Gilpatrick et al., 2019; Hoijer et al., 2018; Stangl et al., 2019; Tsai et al., 2017). The Xdrop technology, on the other hand, requires only nanograms of input DNA, thereby enabling for targeted enrichment of samples where genomic material is limited. Furthermore, it is possible to perform targeted sequencing over larger regions of interest by a tiling design of several Xdrop detection primers. In a CRISPR/Cas9 based protocol, parallel reactions may be required to avoid interference between Cas9 digestions sites tiling the same locus. However, the read lengths of Xdrop enriched DNA are limited by the fragment sizes produced during the dMDA. Currently, the dMDA produces fragments that give average ONT read lengths of 4–5 kb, with some reads over 30 kb (Figure S5). These fragment lengths are sufficient for most applications but might be a limitation if single reads spanning long repetitive regions are required. With improved high molecular weight DNA extraction, optimizations of the dMDA, and possibly size selection of the genomic DNA, we believe it is possible to increase the average fragment lengths. Performing the MDA reaction in droplets has the additional advantage that it reduces the amplification bias between different DNA fragments. Furthermore, the compartmentalization of single DNA molecules reduces the risk of chimeric molecules being generated by the Phi29 polymerase.

While we could detect a significant increase in on‐target reads in the HPV18 enriched HeLa sample as compared to a nonenriched control, a high percentage of reads could not be aligned to HPV18 or flanking genomic regions. It is difficult to determine the cause of this background noise, but we believe it is mainly due to three factors. First, depending on the total DNA amount and the number of droplets, some droplets may contain several DNA fragments originating from different parts of the genome. Even if only one fragment inside a droplet contains the target, all other fragments in the droplet will also be enriched by the FACS sorting. Second, a low fraction of chimeric DNA molecules may be formed during the MDA even though this should be minimized by the compartmentalization into droplets. This could potentially lead to chimeric reads that are difficult to align to the reference genomes. Finally, there is always a risk that a small fraction of negative droplets, not containing the target, end up in the positive fraction after the FACS sorting step. The background noise may be more or less problematic, depending on the specific project. If a higher on‐target percentage is required it may be possible to reduce the background by further optimization of the Xdrop procedure.

Since the method makes it possible to enrich for large unknown flanking genomic regions, it is highly relevant for a wide range of applications including genome gap‐closing (Huddleston et al., 2014), targeted sequencing of hypervariable regions or gene families (Albrecht et al., 2017), investigation of repetitive regions (Loomis et al., 2013) and as demonstrated here in identifying viral insertion sites in a genome. Also, there are several emerging clinical applications of targeted long‐read sequencing (Ameur, Kloosterman, & Hestand, 2019), where the Xdrop technology could prove to be beneficial as compared to alternative enrichment methods. The long fragments generated with the Xdrop method not only lend themselves well to long‐read sequencing but can also provide valuable context information to short‐read sequencing, as demonstrated by the Illumina sequencing results (Table S1).

The described method relies on commercially available instruments, materials, and reagents, which will allow researchers outside specialized microfluid labs to carry out the Xdrop method. In addition, the Xdrop technology has matured and now allows DE droplets to be produced in a single droplet production step. Combined with an automated droplet instrument this will both increase the ease of use, the sample throughput, and reduce the total protocol. With an efficient droplet production and optimization of the final amplification, the procedure can be completed in less than 24 hr from sample to library‐ready enriched sample, and represents an efficient and flexible alternative to hybrid capture or long‐range PCR‐based enrichment methods.

## CONFLICT OF INTERESTS

Esben B. Madsen, Thomas Kvist, and Marie J. Mikkelsen are full time employees at Samplix.

## AUTHOR CONTRIBUTIONS

E. B. M., T. K., and I. H. performed the experiments. E. B. M., I. H., A. A., and M. J. M. wrote the manuscript.

## Supporting information

Supporting information

Supporting information

## Data Availability

The datasets generated and analyzed during the current study is available in the Sequence Read Archive (NCBI) repository under accession: PRJNA596918. https://www.ncbi.nlm.nih.gov/sra/?term=PRJNA596918.
