# Supplementary material for: Xdrop: Targeted sequencing of long DNA molecules from low input samples using droplet sorting
Source: Hum Mutat. 2020 Jun 29;41(9):1671–9. doi: 10.1002/humu.24063 (PMC7496172; doi:10.1002/humu.24063)
Supplement: Supplementary file 2 — Supporting information [file HUMU-41-1671-s002.pdf]

| Template | HPV18 Breakpoint | Human breakpoint, Map position | Human breakpoint, Chr.8 position (GRCh38) | PacBio, Nr Reads | Illumina, Nr Reads | Nanopore, Nr Reads |
|---|---|---|---|---|---|---|
| HeLa | 25 | 8q24 | 127.222.015 | 1 | 97 | 65 |
| HeLa | 2.497 | 8q24 | 127.229.306 | 28 | 55 | 85 |
| HeLa | 3.100 | 8q24 | 127.221.122 | 24 | 3 | 4 |
| HeLa | 5.736 | 8q24 | 127.218.384 | 192 | 157 | 120 |

**Supp. Table S1. Identified HPV18 integrations.**

Position and sequence coverage of HPV18 – Chromosome 8 fusion points detected with PacBio, Illumina and Nanopore sequencing. The positions of the chromosome 8 breakpoint sites refer to the GRCh38 genome assembly

| | Nr Reads | HPV18 reads | Chr.8 fusion region reads | % Chr.8 region reads | % HPV18 reads | Avg. read length | Avg. length, aligned reads |
|---|---|---|---|---|---|---|---|
| ONT +Xdrop | 25092 | 525 | 1088 | 4.3 | 2.1 | 4,317 | 6,113 |
| Illumina +Xdrop | 11171632 | 60558 | 127879 | 1.7 | 0.5 | 126 | 126 |
| PacBio +Xdrop | 41402 | 688 | 2073 | 5.0 | 1.7 | 1543 | 1,774 |
| PacBio gDNA | 294284 | 5 | 5 | 0.003 | 0.0017 | 2042 | 1686 |

**Supp. Table S2. Number of reads, read length and on-target rate.**

Overview of the numbers reads generated, reads mapping to HPV18, reads mapping to chromosome 8 fragile region, percentage on-target reads (HPV18 and chromosome 8 fragile region), percentage HPV18 reads, average read length and average read length of reads mapping to HPV18.

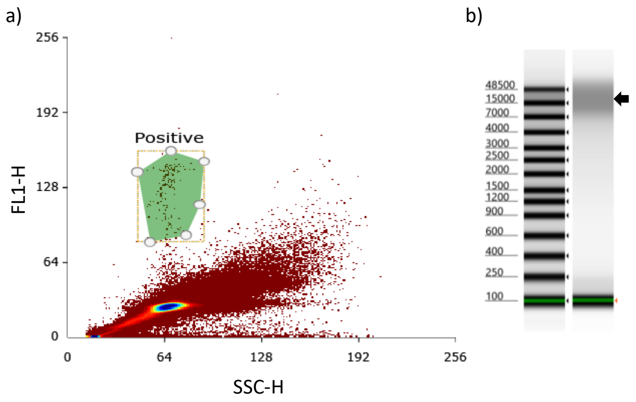


**Supp. Figure S1. A)** PCR-positive droplets are separated from the PCR-negative droplets using side scatter height and FL1 height. **B)** Tapestation DNA fragment length analysis. Lane 1, DNA ladder; lane 2, HPV18 enriched sample after two rounds of dMDA. Black arrow indicates the position and size of the HPV18 enriched sample.

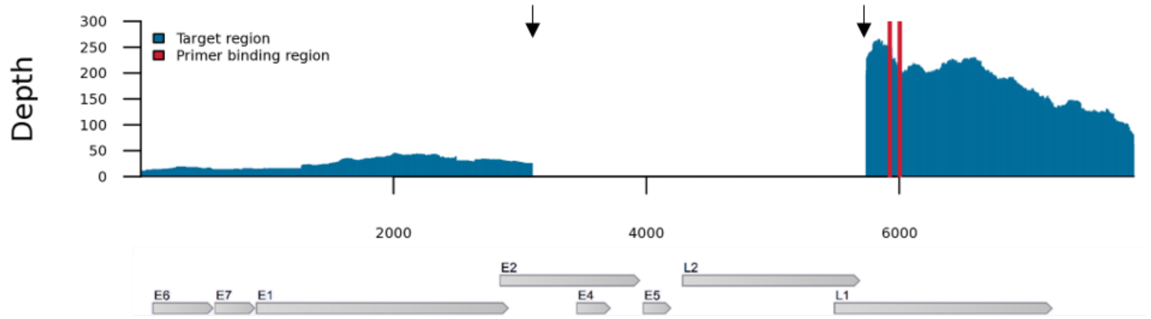


**Supp. Figure S2. HPV18 mapping coverage graph.** 306 PacBio reads were mapped to HPV18 reference genome (NC_001357). The number of reads covering a given position is shown in black. Black arrows indicate the drops in mapping coverage. The location of the HPV18 droplet PCR primers is shown above in red. The position of the HPV18 genes are shown below in grey.

**HPV18-Chromosome 8 fusion point: Chr. 8 127.218.384 – HPV18 5.740:**

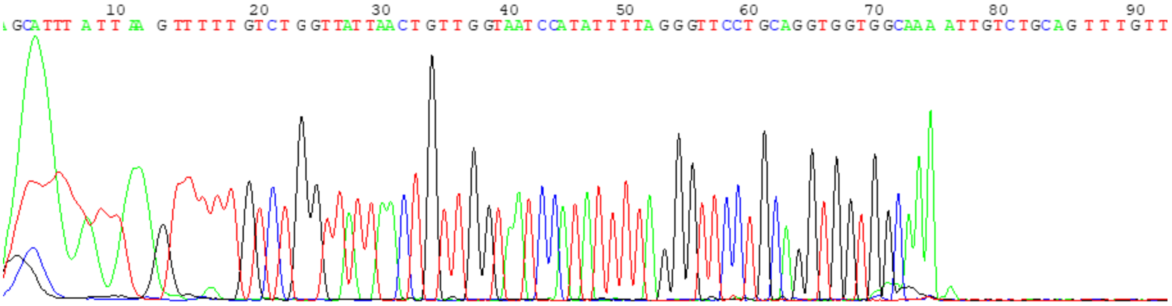

AAGTTTTTGTCTGGTTAT//TAACTGTTGGTAATCCATATTTTAGGGTTCCTGCAGG
TGGTGGCAAA

**HPV18-Chromosome 8 fusion point: Chr. 8 127.221.122 – HPV18 3.100:**

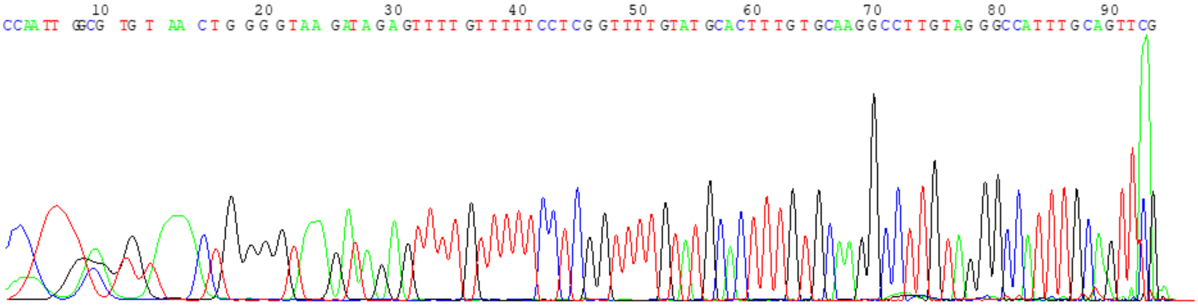

TAACTGGGGTAAGATAGAGTTT//TGTTTTTCCTCGGTTTTGTATGCACTTTGTGCA
AGGCCTTGTAGGGCCATTTGCAGTTCG

**HPV18-Chromosome 8 fusion point: HPV18 22 - Chr8 127.222.015:**

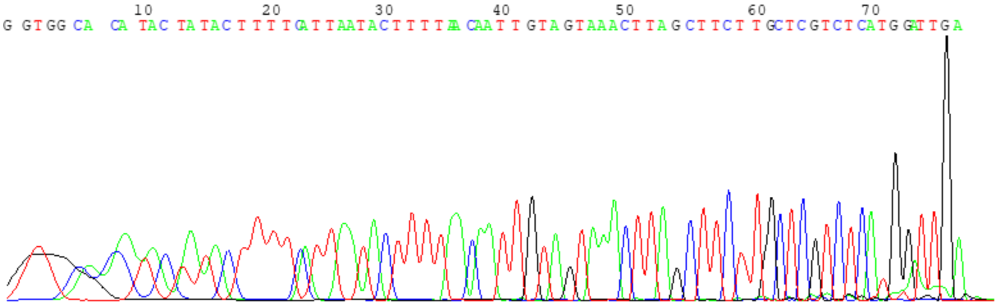

CATACTATACTTTTCATTAATACTTTTAACAATTGTA//GTAAACTTAGCTTCTTGC
TCGTCTCATGGATTGA

**HPV18-Chromosome 8 fusion point: Chr. 8 127.229.306 – HPV18 2.496:**

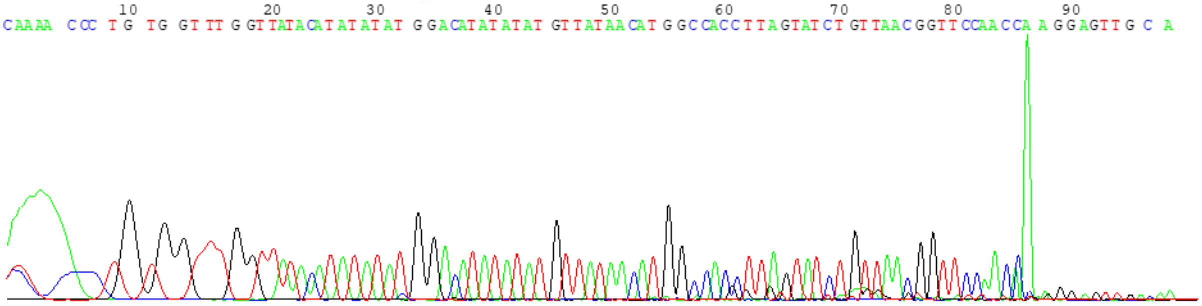

TGTGGTTTGGTTATACATATATATGGACATATATATGTTAT//AACATGGCCACCTT
AGTATCTGTTAACGGTTCCAACC

**Supp. Figure S3. Validation of HPV18-Chromosome 8 fusion points by PCR and Sanger Sequencing.**

Chromatograms and sequence of the four HPV18-Chromosome 8 fusion points. The breakpoint is marked with // in the sequence.

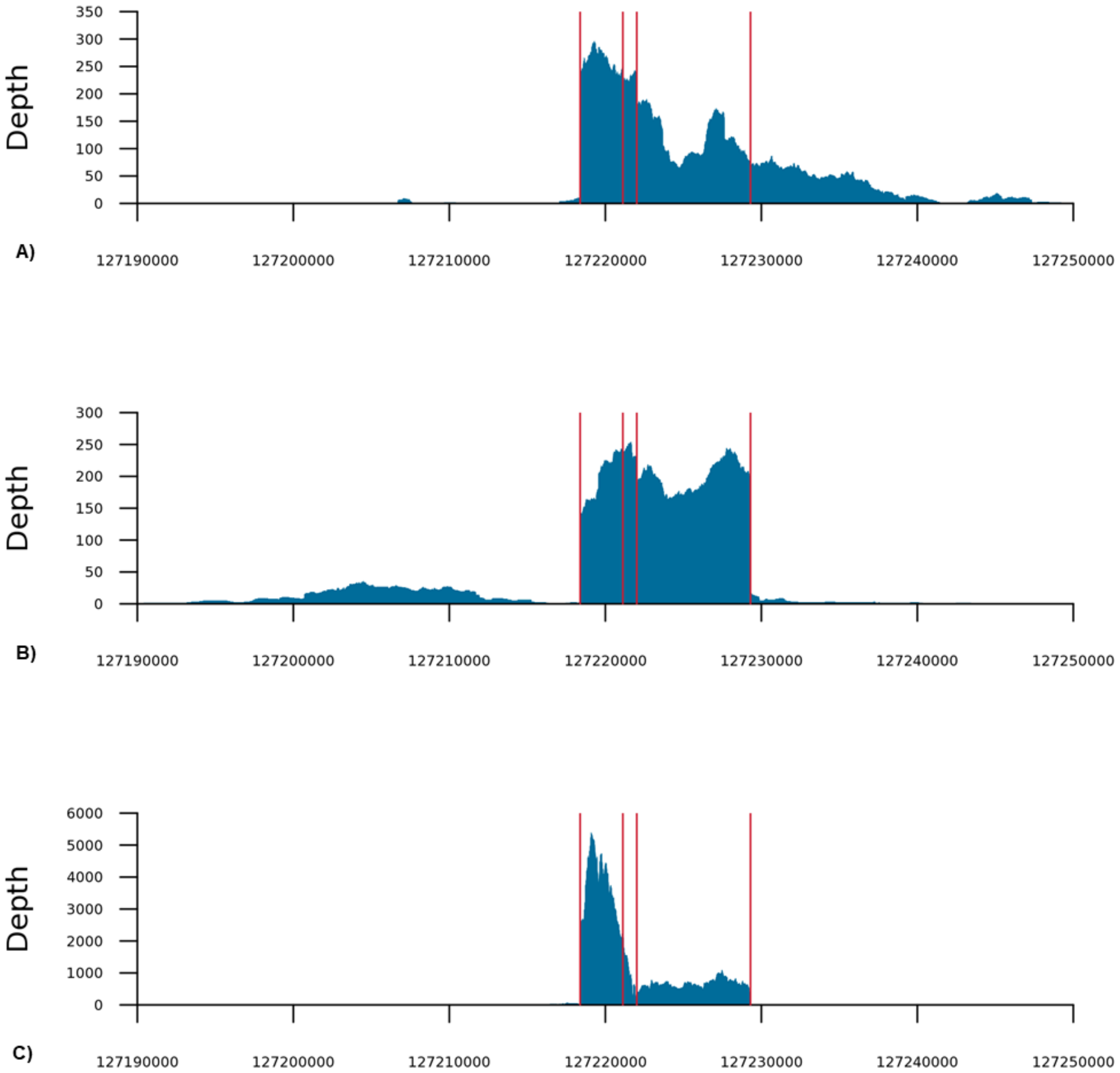


**Supp. Figure S4. Coverage map of chromosome 8 integration site region.** Coverage graph with PacBio data set A) and Oxford Nanopore data set B) and Illumina data set C). The position of the identified HPV18-Chr. 8 fusion points is marked with red vertical lines.

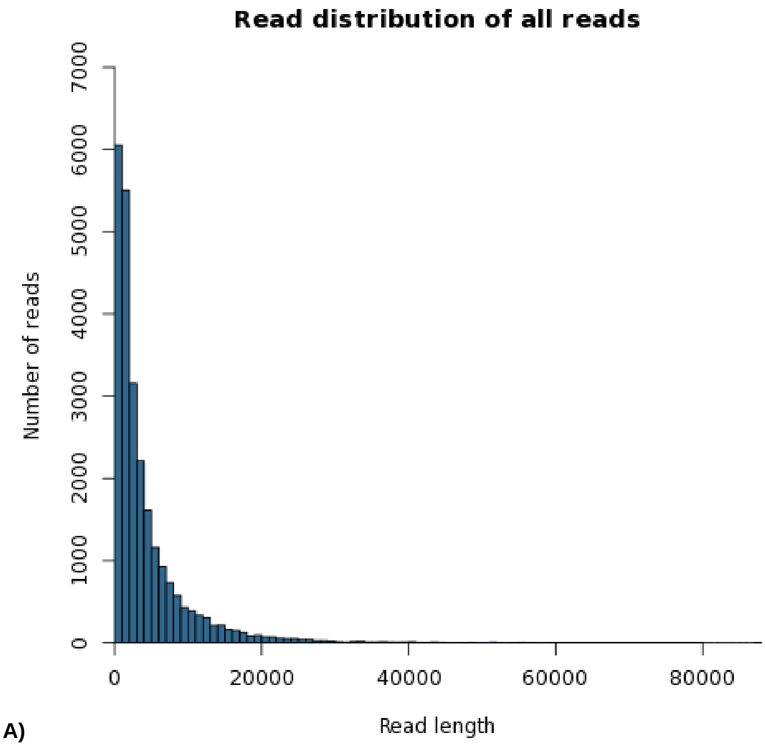


A)

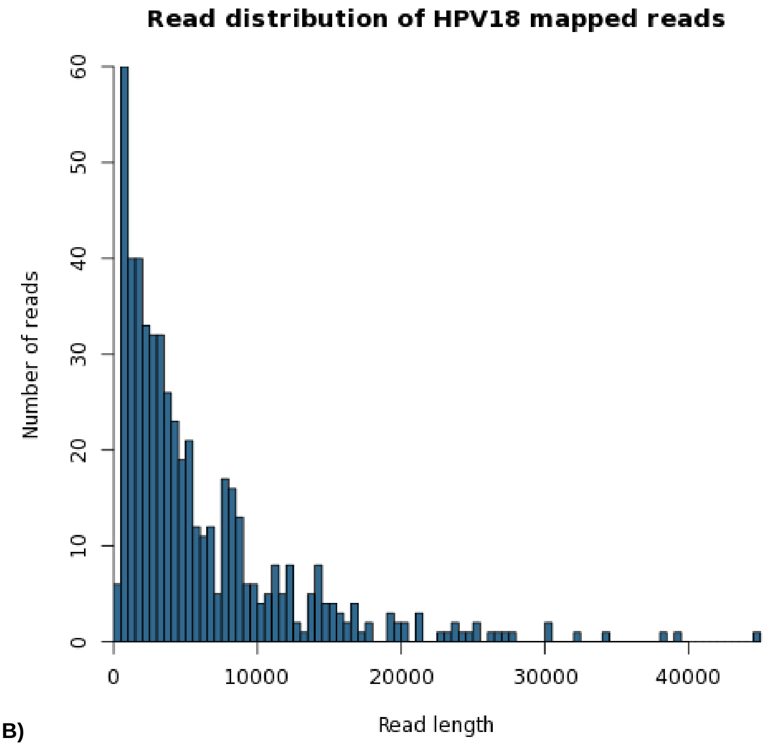


B)

**Supp. Figure S5. ONT read length distribution.** Read distribution of all reads A) and HPV18 mapped reads B)
